# Fetal development and the air pollution exposome: an integrative perspective of health pathways

**DOI:** 10.3389/fncel.2025.1688437

**Published:** 2025-11-25

**Authors:** Eric Alonso Abarca-Castro, José Javier Reyes-Lagos, Kioko Guzmán Ramos, Augusto J. Montiel-Castro, Hypatia Arano-Varela, Pablo Adolfo Mayer-Villa, José Eleazar Aguilar-Toalá, José Luis Montesillo-Cedillo, Ana Karen Talavera-Peña

**Affiliations:** 1 Biological and Health Sciences Division, Autonomous Metropolitan University-Lerma (UAM-L), Lerma, Mexico; 2 Department of Electrical Engineering, Bioelectronics Section, Center for Research and Advanced Studies of the National Polytechnic Institute (Cinvestav), Mexico City, Mexico; 3 Multidisciplinary Research Center in Education, Autonomous University of the State of Mexico, Toluca, Mexico

**Keywords:** exposome, fetal development, air pollution, neurodevelopment, interdisciplinary

## Abstract

We offer an integrative perspective on how the air-pollution exposome shapes fetal development during the first 1,000 days and reverberates across mental health and behavior. Pregnant individuals and young children are disproportionately exposed to particulate matter (PM2.5), nitrogen dioxide (NO_2_), ozone (O_3_), and volatile organic compounds (VOCs) with social disadvantage amplifying risk. We bridge exposure to biology through three conduits. First, the placenta acts as a sensor and recorder, transducing signals that alter growth, immune tone, and neuroendocrine programming. Second, fetal autonomic control–captured by beat-to-beat fetal heart rate variability (fHRV) offers a relevant biomarker of neurodevelopmental integrity; the absence of direct ambient-pollution–fHRV studies is a pressing gap. Third, maternal immune activation, oxidative and endoplasmic reticulum (ER) stress, and disrupted morphogenesis reshape developing circuits, changes now traceable in utero by advanced fetal MRI. These pathways fit a developmental-programming frame: epigenetic remodeling, gene–environment interplay, endocrine-disrupting co-exposures, and gut-microbiome shifts create durable susceptibility. Clinically, the result is structural and functional brain alterations and child phenotypes spanning attention, executive control, affecting regulation, and learning, with clear pediatric and educational implications. We propose an exposome-based research agenda coupling high-resolution exposure assessment with placental molecular profiling, fetal/neonatal autonomic biomarkers (including fHRV), fetal/child neuroimaging, and longitudinal microbiome readouts in harmonized cohorts. In parallel, multisectoral actions–clean air urban design, targeted protection of pregnancy and early childhood, chemical regulation, and risk communication–should narrow exposure inequities while trials test biomarker-guided prevention. Aligning placental biology, autonomic metrics, and exposome science may transform risk stratification and safeguard the developing brain.

## 1 Introduction

This perspective provides an interdisciplinary analysis of how air pollution, including particulate matter (PM2.5), nitrogen dioxide (NO_2_), ozone (O_3_), and volatile organic compounds (VOCs) affects fetal health at the molecular and cellular levels, subsequently influencing mental health, cognitive development, neurodevelopment, and social behavior (Inoue et al., 2020; Veras and Saldiva, 2025). The first 1,000 days of life, encompassing the prenatal period, represent a uniquely sensitive window during which environmental factors, such as air pollution, could profoundly shape neurodevelopmental trajectories due to dynamic processes like neurogenesis, synaptic pruning, and myelination (Costa et al., 2017; Bové et al., 2019).

The exposome broadens the traditional perspective on environmental risks by considering the cumulative effect of all internal and external exposures on individual experiences from conception to the end of life. Originally defined by Wild (2005) as the total set of exposures from conception to death, the exposome includes not only environmental pollutants (e.g., air pollution) but also diet, psychosocial stress, infections, lifestyle, medications, and social and economic environments (Vineis et al., 2020).

The exposome encompasses three broad categories. The general external exposome includes socioeconomic conditions, educational level, urbanization, and the social environment. The specific external exposome refers to more direct exposures, such as air pollution, diet, physical activity, and infections. The internal exposome involves endogenous biological processes, including inflammation, oxidative stress, metabolism, the microbiota, and biomarkers of exposure (Baluch et al., 2020). During the first 1,000 days, this approach is especially relevant, as the organism is more vulnerable and exposures may have disproportionate and lasting effects on neurodevelopment, enabling the identification of interactions between environmental, social, and biological factors and supporting timely prevention and intervention (Vrijheid et al., 2011; Sun et al., 2025). Notably, recent evidence underscores that air pollution is a critical public health threat, disproportionately affecting vulnerable populations, including pregnant individuals and young children, particularly in urban and socioeconomically disadvantaged contexts (Vrijheid et al., 2011; World Health Organization, 2021).

We first synthesize the environmental burden and exposure pathways in pregnancy and early childhood (Section “2 Environmental burden and exposure pathways in pregnancy and early childhood”). We then present mechanistic bridges from exposure to biology, highlighting the placenta as a sensor-recorder and fetal autonomic regulation via fetal heart rate variability or fHRV (Section “3 Mechanistic bridges: from exposure to biology via the placenta, fetal physiology, and the developing brain”). Next, we examine developmental programming through epigenetics, gene—environment interplay, and endocrine-disruptive chemicals (EDC) mixtures [Section “4 Epigenetics and developmental programming (G × E and EDCs)”]. We then summarize observable child phenotypes across structural and functional domains (Section “5 Clinical psychology, pediatrics, and child development: observable phenotypes”). Finally, we propose an exposome-based framework for research and policy (Section “6 An exposome-based framework for research and policy”) and close with a call for interdisciplinary action (Section “7 Conclusions and call for interdisciplinary action”).

## 2 Environmental burden and exposure pathways in pregnancy and early childhood

This subsection summarizes the population burden of air-pollution exposure in pregnancy/early childhood and clarifies the main pathways by which these exposures reach and affect the mother–placenta–fetus unit.

Environmental medicine data indicate that pregnant individuals and young children are routinely exposed to harmful levels of atmospheric pollutants such as PM2.5, NO_2_, O_3_, and VOCs particularly in urban environments and among socioeconomically disadvantaged populations (Vrijheid et al., 2011; World Health Organization, 2021). These exposures frequently exceed international safety guidelines and are associated with an increased risk of adverse birth outcomes, including low birth weight, preterm birth, and congenital anomalies (Ravindra et al., 2021; Desye et al., 2024); from an epidemiological perspective, these risks are determinants of health that influence both exposure levels and resilience to harm. Indeed, these social determinants can outweigh the influence of genetic factors or even healthcare access in shaping health outcomes (Conway et al., 2024; Veras and Saldiva, 2025; World Health Organization, 2025). According to the World Health Organization (WHO) Global Air Quality Guidelines (2021), concentrations above specific thresholds are considered harmful to human health: PM2.5 should not exceed 5 μg/m^3^ as an annual mean and 15 μg/m^3^ as a 24-h mean; PM10, 15 μg/m^3^ annually and 45 μg/m^3^ daily; NO_2_, 10 μg/m^3^ annually and 25 μg/m^3^ daily; O_3_, 100 μg/m^3^ as an 8-h mean; and SO_2_, 40 μg/m^3^ as a 24-h mean. These reference values provide the necessary context to interpret statements that pollutant concentrations “exceed international safety guidelines.” Importantly, WHO emphasizes that there is no safe threshold for fine particulate matter (PM2.5), as adverse health outcomes–including cardiovascular, respiratory, and neurodevelopmental effects–have been documented even below these guideline levels (World Health Organization, 2021).

From an epidemiological perspective, these risks are determinants of health that influence both exposure levels and resilience to harm. Indeed, social and structural determinants–such as poverty, housing conditions, and access to healthcare–can outweigh the influence of genetic factors or individual behavior in shaping health outcomes (World Health Organization, 2025).

Consistently, epidemiological studies have robustly linked prenatal exposure to elevated pollutant levels with prematurity and low birth weight, which are themselves risk factors for long-term neurodevelopmental disorders like attention deficit hyperactivity disorder (ADHD) and autism spectrum disorder (ASD) (Melody et al., 2019; Hu et al., 2024).

It is essential to understand the main exposure pathways during pregnancy and early childhood. While inhalation of fine particles is the most extensively studied route, exposure may also occur through ingestion of contaminated water or food, or via the transplacental transfer of lipophilic pollutants that accumulate in maternal tissues (Veras and Saldiva, 2025). The placenta, far from being a completely protective barrier, can allow the passage of contaminants that impair fetal growth and alter the programming of developing organs and systems (Vrijheid et al., 2011).

Another critical aspect is the specific biological vulnerability of these early developmental stages. During gestation and early childhood, the respiratory, cardiovascular, nervous, and immune systems are still developing and are more susceptible to alterations induced by environmental pollutants (World Health Organization, 2021). This heightened sensitivity can lead to low-grade chronic inflammation, oxidative stress, and endothelial dysfunction, with potential long-term health repercussions (Veras and Saldiva, 2025).

Beyond direct organ effects, the gut microbiome has emerged as an additional pathway of impact and potential biomarker of exposure. Atmospheric pollutants can indirectly affect health by modulating gut microbial composition and diversity, a key determinant of wellness and disease (Manor et al., 2020). For example, both acute and chronic exposure to NO_2_ immediately after birth and during early life have been associated with reduced gut microbial diversity (Cruells et al., 2024), one of several relevant indicators of well-being (de Vries et al., 2022). Growing evidence suggests that exposure to fine particulate matter (PM2.5, black carbon) can alter the gut microbiota, with decreases in the bacterial phylum Bacteroidetes and increases in Proteobacteria (Li et al., 2023), the latter being associated with bacterial dysbiosis (Rizzatti et al., 2017). Imbalances in the early-life gut microbiome have been linked to systemic diseases (e.g., diabetes, asthma, enteric inflammation) later in life (Isolauri, 2012). When detected in population-based studies, these microbiota changes can serve as early biomarkers of environmental exposure and may contribute to maternal and child health risk stratification.

## 3 Mechanistic bridges: from exposure to biology via the placenta, fetal physiology, and the developing brain

The placenta is a transient but highly specialized organ that suppresses maternal immune rejection and transports oxygen, nutrients, hormones, and growth factors to the fetus while exporting waste products in the opposite direction; these functions depend on a multilayered barrier of trophoblasts, endothelial cells, and Hofbauer macrophages (Levkovitz et al., 2013; Burton et al., 2014). Its endocrine output and bidirectional signaling make it not merely a passive barrier a first responder to environmental toxicants that reach maternal blood, positioning placental tissue as both a target and a recorder of air-pollution exposure (Janssen et al., 2013; Nugent and Bale, 2015).

Traditional fetal surveillance hinges on mean fetal heart rate (FHR), yet a registry analysis of 23,782 pregnancies showed that a 10.7 μg/m^3^ rise in first-trimester PM2.5 increased false-positive FHR alarms by 20% without biochemical acidosis, suggesting pollutant-induced alterations in fetal cardiac reactivity (Morokuma et al., 2017). Beat-to-beat fetal heart rate variability (fHRV) captures the maturation of sympathetic and parasympathetic control and may therefore offer a more sensitive marker of neurodevelopmental integrity (DiPietro et al., 2007). Our literature scan found no studies directly linking ambient air pollution with fHRV, highlighting a critical evidence gap. Tobacco exposure provides proof of concept: in 6,491 late-gestation fetuses, continued maternal smoking reduced mean FHR, dampened movement, and disrupted HR–movement coupling in a dose-dependent fashion (Lucchini et al., 2021). Follow-up data in preschool children prenatally exposed to nicotine via smokeless tobacco revealed higher systolic blood pressure and a shifted low-frequency to high-frequency HRV ratio, implicating persistent autonomic imbalance (Nordenstam et al., 2019). Collectively, these findings indicate that integrating fHRV with placental molecular profiling in birth cohorts could sharpen risk assessment, reduce obstetric diagnostic noise, and illuminate mechanistic links between early pollutant exposure and long-term neuro-cardiovascular health. Taken together, these observations support a coherent pathway from exposure → placental signaling → fetal autonomic regulation → structural and functional brain changes.

Neurobiologists emphasize the developing brain’s susceptibility to environmental insults. Air pollutants trigger maternal immune activation, elevating pro-inflammatory cytokines that cross the placenta and disrupt fetal neurodevelopment (Block and Calderón-Garcidueñas, 2009). Animal models demonstrate that prenatal exposure to air pollutants impairs synaptic plasticity, disrupts myelination, and induces structural changes in critical brain regions such as the cortex and hippocampus, which are essential for emotional regulation and cognitive functioning (Bolton et al., 2017).

Recent advances show that the air-pollution exposome affects the developing brain through structural and cellular pathways. High-resolution fetal MRI now links mid-gestation exposure to NO_2_, PM2.5, and black carbon with a 4%–8% reduction in cortical surface area, delayed cerebellar gyrification, and expanded cerebrospinal fluid spaces by 32 weeks’ gestation–indicating teratogenic effects manifest in utero rather than only in childhood follow-ups (Gómez-Herrera et al., 2025). At the cellular level, in animal models, gestational PM2.5 triggers oxidative and endoplasmic-reticulum stress in the hypothalamus, down-regulates tyrosine hydroxylase, and produces depressive-like behavior, implicating disrupted dopaminergic signaling (Kim et al., 2024). Additionally, gestational PM2.5 selectively down-regulates the transcription factor homeobox A5 (HOXA5), stunts axonal and dendritic growth, and produces male-biased spatial-memory deficits, linking particulate exposure to disrupted neuronal morphogenesis (Hou et al., 2023). Functionally, the behavioral toll of prenatal air pollution has been documented in Mexico City’s longitudinal PROGRESS cohort: a 6 μg/m^3^ interquartile increment in PM2.5 during the second or third trimester raised the odds of belonging to the “low inhibitory-control” class on Go/No-Go tasks at 9–10 years by ∼60% (Bansal et al., 2021), pinpointing a window that overlaps rapid axonal outgrowth and synaptic pruning.

At the cellular and molecular level, ambient PM2.5/NO_2_/O_3_/VOCs induce oxidative and endoplasmic-reticulum stress and epigenetic remodeling, with carbonaceous particles crossing the placenta and localizing in fetal organs, and gestational PM2.5 perturbing hypothalamic development, thereby linking exposure to placental and fetal-brain targets (Gruzieva et al., 2019; Bongaerts et al., 2022; Kim et al., 2024).

## 4 Epigenetics and developmental programming (G × E and EDCs)

Growing evidence from epigenetic research highlights the marked sensitivity of fetal development to environmental stressors, particularly airborne pollutants (Perera and Herbstman, 2011). Environmental evidence indicates that pregnant individuals and young children are constantly exposed to PM2.5, NO_2_, O_3_, VOCs, and EDCs at levels that often exceed safety guidelines (Vrijheid et al., 2014; World Health Organization, 2021). Epigenetic research reveals air-pollution-induced changes in DNA methylation, histone modifications, and non-coding RNA expression in the placenta and fetal tissue (Janssen et al., 2013; Breton et al., 2016). These epigenetic alterations affect gene expression patterns crucial for neural development, stress response, and immune function, potentially mediating long-term behavioral and cognitive outcomes in line with developmental origins of health and disease principles (Ho et al., 2012). Importantly, placental epigenetic marks can serve as an exposure “record” that pairs naturally with autonomic readouts (e.g., fHRV) to assess exposure–biology links.

Building upon this epigenetic perspective, gene–environment interaction studies have found that the interplay between specific genotypes (e.g., GSTM1 null, OGG1 null, H2AX AG/GG) and environmental factors (e.g., cereal consumption, in-house cockroaches, home crowding, humidity during the first year of life) with PM2.5 is associated with increased DNA damage (Marín et al., 2024). This evidence illustrates the complex interplay between genetic background and environmental exposure in shaping early-life health outcomes.

Endocrine-disrupting chemicals are compounds that alter hormone metabolism, signaling, and homeostasis of the endocrine system and can modify gene expression (Braun, 2017). EDCs include volatile or semi-volatile molecules commonly detected in dust and in indoor/outdoor air (Rudel and Perovich, 2009). In addition to EDCs, the above-mentioned airborne pollutants (PM2.5, NO_2_, O_3_, VOCs) –many with endocrine-disrupting properties– are widespread and tend to co-occur in complex mixtures. Fetuses, neonates, and infants are particularly susceptible due to multiple exposure pathways, including transplacental and lactational transfer, inhalation, and ingestion of contaminated dust, food, and beverage packages; their heightened vulnerability is compounded by immature detoxification systems and distinct toxicokinetic processes. Disruption of hormonally regulated, time-sensitive developmental processes and epigenetic pathways during early life may significantly increase the risk of neurodevelopmental disorders and obesity (Rudel and Perovich, 2009; Braun, 2017).

Studies using human biological models have linked the presence of EDCs in placental tissue with altered gene-expression profiles mediated by epigenetic modifications–such as global and locus-specific DNA hypomethylation–and telomere shortening in placental and umbilical cord cells. These molecular alterations associate with adverse outcomes including preterm birth, restricted fetal growth and development, thyroid dysfunction (physiological thyroid hormone levels are fundamental for normal neurodevelopment), neurological disorders, and increased risk of subsequent metabolic diseases (Basak et al., 2020; Isaevska et al., 2021). Moreover, prenatal and early postnatal exposure to EDCs and air pollutants may contribute to behavioral disorders and cognitive deficits in children (Rudel and Perovich, 2009; Braun, 2017). Together, these lines of evidence position developmental programming as a plausible mediator between the air-pollution exposome and later neurobehavioral phenotypes.

## 5 Clinical psychology, pediatrics, and child development: observable phenotypes

This subsection summarizes structural and functional phenotypes observed in children following prenatal/early-life exposure to air pollution. The association between prenatal exposure to air pollutants and developmental difficulties is well documented. Structural brain differences include reductions in white matter (Peterson et al., 2015; de Prado Bert et al., 2018; Herting et al., 2019), cortical gray matter (de Prado Bert et al., 2018; Herting et al., 2019), basal ganglia (de Prado Bert et al., 2018), and caudate nucleus (Herting et al., 2019). Functional difficulties have been reported in adaptive skills, social skills, and adaptive communication (McGuinn et al., 2020); in memory functioning and attention (Mathilda Chiu et al., 2023); in language and fine/gross motor domains; and in global intelligence quotient (Suades-González et al., 2015). In clinical populations, higher severity of ASD symptoms and an increased risk of ADHD have also been reported (Thygesen et al., 2020; Zhao et al., 2024).

Clinical psychologists further highlight links between prenatal and early-life air-pollution exposure and behavioral/emotional challenges, including heightened anxiety, mood disorders, and attentional deficits (Perera et al., 2013; Peterson et al., 2015). These outcomes may result from pollution-induced neurotoxic effects or indirect impacts on regulatory brain regions–prefrontal cortex, anterior cingulate, and limbic structures–as well as environmental modifiers such as family stress and reduced outdoor activities.

Pediatricians emphasize the cumulative impact of pollution exposure on child health and neurodevelopment. Beyond respiratory illness, compromised neurodevelopment affects academic achievement, social integration, and long-term health (Sunyer et al., 2015; Chong-Neto and Filho, 2025). Early interventions hold the potential for significant health and societal benefits, as shown by improved child neurodevelopment after the closure of a coal-fired power plant that reduced prenatal PAH exposure (Frederica et al., 2008).

## 6 An exposome-based framework for research and policy

Building on the foregoing mechanisms, understanding the multifaceted ways in which the air-pollution exposome shapes fetal and early childhood development requires a framework that seamlessly integrates environmental science, biomedical research, and psychosocial context. During the critical first 1,000 days of life, exposures to PM2.5, NO_2_, O_3_, VOCs, and mixtures of EDCs often occur in synergy with social disadvantage, amplifying biological vulnerability.

**FIGURE 1:**
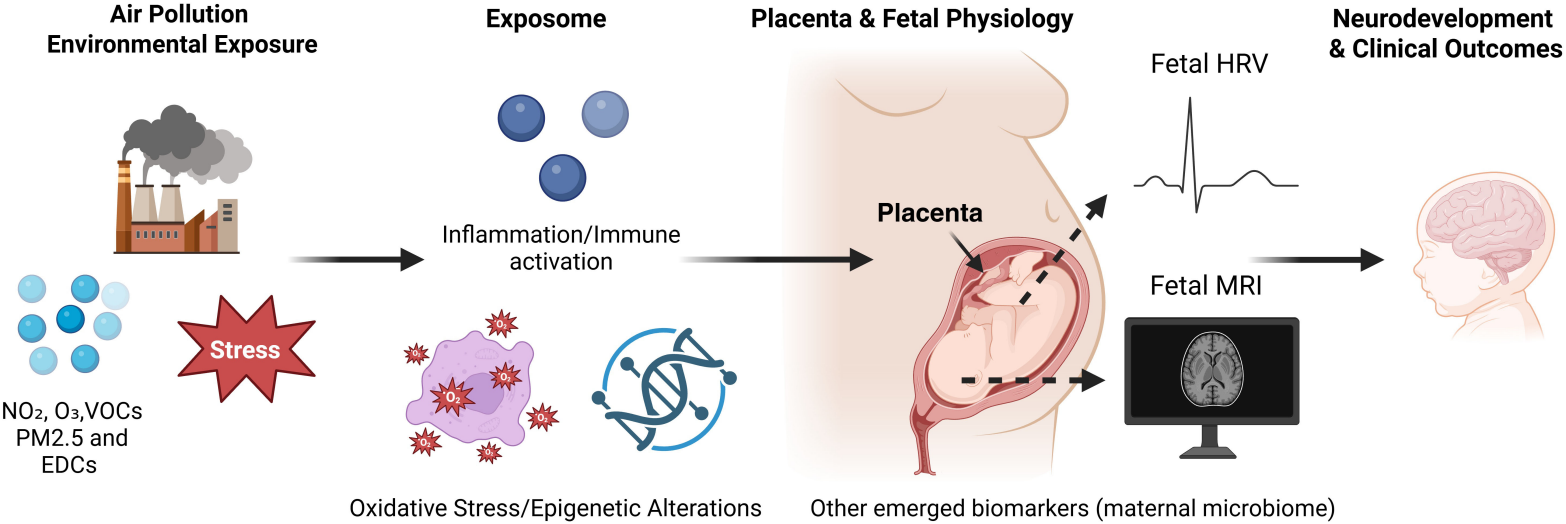
Conceptual model linking air pollution exposure to fetal neurodevelopment. Maternal exposure to environmental pollutants such as nitrogen dioxide (NO_2_), ozone (O_3_), volatile organic compounds (VOCs), fine particulate matter (PM2.5), and endocrine-disrupting chemicals (EDCs) triggers systemic stress responses. These exposures contribute to oxidative stress, immune activation, inflammation, and epigenetic alterations, summarized within the exposome framework. Such biological processes may affect placental function and fetal physiology. Emerging approaches, including assessment of fetal heart rate variability (fHRV), fetal magnetic resonance imaging (MRI), and other novel biomarkers (e.g., maternal microbiome), provide windows into in utero adaptations. Ultimately, these pathways may shape neurodevelopmental trajectories and clinical outcomes in early life (e.g., Autism Spectrum Disorder, Attention Deficit/Hyperactivity Disorder).

The placenta functions as a dynamic biosensor and mediator, translating maternal exposures into molecular, endocrine, and immune signals that influence growth, immune tone, and neurodevelopmental programming. Fetal autonomic regulation, captured through high-resolution measures such as beat-to-beat fHRV, offers a sensitive yet underutilized biomarker of neurodevelopmental integrity, while advanced neuroimaging reveals that pollutant-induced structural and functional brain changes can manifest in utero (Figure 1).

These processes are further shaped by epigenetic remodeling, gut microbiome alterations, and complex gene–environment interactions, establishing trajectories of susceptibility that may persist across the lifespan. An exposome-based approach facilitates the integration of multimodal exposure assessment with placental molecular profiling, autonomic biomarkers, neuroimaging, and microbiome metrics in harmonized, longitudinal birth cohorts, enabling spatial epidemiology, risk mapping, and precision prevention strategies. Translating this science into policy demands coordinated, multisectoral engagement between environmental agencies, health systems, urban planners, and communities, with measures such as clean-air zones, reduction of traffic emissions near homes and schools, expansion of urban green spaces, and maternal education programs to reduce indoor exposures. Embedding these strategies within an exposome framework bridges mechanistic insights with actionable public health interventions, ensuring that the protection of neurodevelopment is recognized as both a public health priority and a social imperative.

## 7 Conclusions and call for interdisciplinary action

Air pollution during the first 1,000 days intersects with biological vulnerability to reshape neurodevelopmental trajectories via placental sensing, neuroimmune activation, autonomic and neural circuit maturation, and epigenetic programming. An integrated exposome framework, implemented through interdisciplinary collaboration among environmental medicine specialists, epidemiologists, neurobiologists, epigeneticists, clinical psychologists, pediatricians, and public health professionals, can improve risk assessment, guide evidence-based interventions, and inform effective policies to safeguard the neurodevelopment and health of future generations.

Moving forward, our collective challenge is to operationalize this framework within harmonized, longitudinal birth cohorts that allow real-time integration of exposure data, biological signatures, and developmental outcomes. Embedding such science in policy will require not only technological and methodological innovation but also sustained multisectoral commitment to environmental justice, ensuring that protection of neurodevelopment is recognized as both a public health priority and a social imperative.

## Data Availability

The original contributions presented in this study are included in this article/supplementary material, further inquiries can be directed to the corresponding author.
